# The Alarmin Concept Applied to Human Renal Transplantation: Evidence for a Differential Implication of HMGB1 and IL-33

**DOI:** 10.1371/journal.pone.0088742

**Published:** 2014-02-20

**Authors:** Antoine Thierry, Sébastien Giraud, Aurélie Robin, Anne Barra, Franck Bridoux, Virginie Ameteau, Thierry Hauet, Jean-Philippe Girard, Guy Touchard, Jean-Marc Gombert, André Herbelin

**Affiliations:** 1 Service de Néphrologie-Hémodialyse-Transplantation rénale, Centre Hospitalier Universitaire de Poitiers, Poitiers, France; 2 Institut national de la santé et de la recherche médicale U1082, Poitiers, France; 3 Université de Poitiers, Poitiers, France; 4 Institut national de la santé et de la recherche médicale U935, Poitiers, France; 5 Laboratoire d’Immunologie, Centre Hospitalier Universitaire de Poitiers, Poitiers, France; 6 Laboratoire de Biochimie, Centre Hospitalier Universitaire de Poitiers, Poitiers, France; 7 Institut de Pharmacologie et de Biologie Structurale, Toulouse, France; 8 Centre national de la recherche scientifique, Unité Mixte de recherche, Toulouse, France; 9 Université de Toulouse, Toulouse, France; Cordelier Research Center, INSERMU872-Team16, France

## Abstract

The endogenous molecules high mobility group box 1 (HMGB1) and interleukin-33 (IL-33) have been identified as alarmins, capable of mediating danger signals during tissue damage. Here, we address their possible role as innate-immune mediators in ischemia-reperfusion injury (IRI) following human kidney transplantation. We analysed serum and urinary HMGB1 and IL-33 levels, all determined by enzyme-linked immunosorbent assay, in a cohort of 26 deceased renal transplant recipients. Urinary HMGB1 and IL-33 levels were significantly increased as soon as 30 min after reperfusion, as compared to those before treatment. Moreover, both serum and urinary IL-33 (but not HMGB1) increase was positively correlated with cold ischemia time, from 30 min to 3 days post-transplantation. *In vitro*, human umbilical vein endothelial cells subjected to hypoxia conditions released both HMGB-1 and IL-33, while only the latter was further increased upon subsequent re-oxygenation. Finally, we postulate that leukocytes from renal recipient patients are targeted by both HMGB1 and IL-33, as suggested by increased transcription of their respective receptors (TLR2/4 and ST2L) shortly after transplantation. Consistent with this view, we found that iNKT cells, an innate-like T cell subset involved in IRI and targeted by IL-33 but not by HMGB1 was activated 1 hour post-transplantation. Altogether, these results are in keeping with a potential role of IL-33 as an innate-immune mediator during kidney IRI in humans.

## Introduction

Ischemia-reperfusion injury (IRI) contributes to the development of renal graft damage associated with renal transplantation [1]. It has been established that inflammation together with oxidative stress are involved in kidney IRI, resulting in cell necrosis and apoptosis. Such an inflammatory process is independent of donor-specific alloreactivity and involves both innate and adaptive immune responses [2] [3]. Countering the early immune IRI events could be beneficial for long-term allograft survival [4], notably for organs from extended criteria donors, that are more prone to IRI [5]. To this purpose, the mechanisms underlying inflammation mediated by innate immunity during kidney IRI need to be better understood.

Damage-associated molecular pattern (DAMP) molecules, including alarmins, which mediate “danger” signals, leading to the propagation of inflammatory reactions and the initiation of adaptive immune responses are released by cells that have been damaged by IRI [6] [7]. The prototype DAMP, a chromatin-associated non-histone protein, high-mobility group box 1 (HMGB1), is widely expressed in mammalian cells with dual functions as both nuclear factor and as pro-inflammatory cytokine, released from the necrotic cells [8]. HMGB1 is also recognized as an alarmin defined as an endogenous molecule that signals tissue and cell damage by initiating rapidly innate immune responses. It has been implicated in early loss of transplanted islets [9] or in liver IRI [10] in mouse models. Initiating the early inflammatory response, HMGB1 contributes also to kidney IR in mice, while its neutralization protects renal cells from this injury [11] [12]. Both of the most widely studied DAMP receptors, Toll-like receptor 2 (TLR2) and TLR4, whose expression by tubular epithelial cells can be modulated by hypoxia [13] and oxidative stress [14], respectively, are multi-ligand receptors that bind HMGB1. However, while TLR2 and TLR4 mediate kidney IR injury in mice [12] [15], little is known about HMGB1 release and its putative interaction with TLR2 and TLR4 receptors in the context of human kidney transplantation [16].

The recently identified IL-33 is the ligand of ST2L, a member of the IL-1 receptor superfamily [17]. Its signalling pathway can be negatively regulated by sST2 (the soluble form of ST2) that potentially acts as a decoy receptor for IL-33 [18]. Since its first description, important roles have been ascribed to IL-33, both as a conventional cytokine and as an alarmin [19] [20]. Recently, IL-33 has been implicated in acute and chronic kidney diseases in humans [21] [22]. In mice, Sakai *et al*. reported that IL-33 and ST2L expression was increased early after hepatic IR injury, suggesting that IL-33 may act as an endogenous regulator of liver IR [23]. However, a role of the IL-33/ST2L axis has not previously been described in IRI after kidney transplantation. Knowing on the one hand that endothelial cells and tubular epithelial cells are potential sources of IL-33 [19] and on the other hand that IL-33 is released passively in active forms into the intercellular milieu during necrosis to target the innate immune response, we hypothesized that IL-33 might play a part in renal IRI.

In the present study, we addressed the role of both HMGB1/TLR and IL-33/ST2 alarmin pathways early after human kidney transplantation. Our findings support the concept that IL-33 and HMGB1 act during kidney IR as alarmins whose release is induced *in vitro* by hypoxia and/or re-oxygenation conditions as well as *in vivo* in serum and/or urine promptly after reperfusion. Remarkably, a correlation between IL-33 (but not HMGB1) levels and IRI duration was found, thus providing evidence for a close association between cell injury and IL-33 release.

## Materials and Methods

### Patients

A prospective cohort study was conducted on 26 consecutive kidney transplant patients at the Transplant Unit of the University Hospital of Poitiers between December 1, 2009 and October 30, 2010. Adult recipients (18–70 years) of a first (n = 22) or second kidney transplant (n = 5) were eligible for enrollment. Among these, 7 patients (31%) were pre-emptively transplanted (Table 1). Analysis of HMGB1, IL-33 and sST2 serum levels before kidney transplantation showed no difference between pre-emptively and non-pre-emptively transplanted patients (data not shown).

**Table 1:** Baseline demographic and clinical characteristics of recipients and donors.

Variables		n = 26
**Recipient characteristics**		
Age (year)		51.4±5.2
Gender	Male	17 (62.9%)
BMI(1)		24.80±4.3
Initial nephropathy	Polycystic kidney	7
	Glomerulopathy	8
	Nephroangiosclerosis	2
	Other	9
Residual diuresis (%)		20 (77%)
**Donor characteristics**		
Age (year)		51.0±10.1
Gender (%)	Male	14 (53.8%)
Cause of death (%)	Cardio vascular accident	17
	Brain trauma	5
	Other	4
ECD(2) (%)		11 (42%)
**Transplant characteristics**		
Pre-emptive transplantation		7 (31%)
Cold ischemia time (h)		14,9±4,2
Warm ischemia time (min)		42,4±14,9
Human HLA antibodies	No	58%
	Yes	42%
Total HLA mismatch (n)		5.0±1.5
CMV status (%)	D+/R−	11.5%
	D−/R+	19.2%
	D+/R+	30.8%
	D−/R−	38.5%
DGF(3) (%)		2 (7.7%)

(1) BMI: Body Mass Index.

(2) ECD: Expanded Criteria Donors.

(3) DGF: Delayed Graft Function defined as a need for dialysis within the first week after transplantation.

All recipients received immunosuppressive treatment based on an induction therapy (either basiliximab or Thymoglobulin), a calcineurin inhibitor (either cyclosporine or tacrolimus) and a secondary agent (either mycophenolate mofetil or enteric-coated mycophenolate sodium). The steroid was administered at a dose of 500 mg on day 0, and at 0.5 mg/kg/day from day 1 to day 7 post-transplantation, followed by a progressive decrease.

Renal function was assessed by estimated glomerular filtration rate (eGFR) according to the simplified modification of diet in renal disease formula. Mean (± SD) eGFR was 43.5±3.9 mL/min/1.73 m^2^, 45.9±3.4 mL/min/1.73 m^2^ and 49.5±3.5 mL/min/1.73 m^2^ at 1, 3 and 12 months post-transplantation, respectively. The incidence of biopsy proven acute rejection during the first year was 26.9%. There was no patient death and only one graft loss during the study period.

### Ethics Statement

The blood samples were taken during the normal follow-up of the patient, anonymized and since the study did not require additional blood sampling, an approval from an ethics committee was not required under French law according to the article L.1121-1 of the public health code. The article states that: The research organized and performed on human beings in the development of biological knowledge and medical research are permitted under the conditions laid down in this book and are hereinafter referred to by the term “biomedical research”. The article further states that it does not imply under conditions: “For research in which all actions are performed and products used in the usual way, without any additional or unusual diagnostic procedure or surveillance.” Written informed consents were obtained from each patient according to the Declaration of Helsinki. Collection of blood samples from anonymized hematopoietic stem cells donors (Hematology Unit of the University Hospital of Poitiers, France), defined in this study as healthy individuals, was performed according to strict protocols approved by ethical policy of Poitiers University Hospital, including written informed consent prior to participation in the study.

### Sample Collection

Peripheral blood was collected by venepuncture at day 0 (D0) before kidney transplantation and within 0.5 hour (h) (H0.5) and 3 h (H3) from the recipients after kidney transplantation, typically on transfer to the post-anesthesia unit and in the morning of the first and third post-operative day (POD). Urine samples were collected at the same time points and centrifuged at 5000 g to remove cellular debris. For flow cytometry and mRNA analysis, blood was recovered in tubes containing heparin as an anticoagulant. For serum samples, blood was collected without anticoagulant. PBMCs were separated by performing a density gradient centrifugation on Ficoll*-*Paque and cryopreserved prior flow cytometry analysis. Blood leukocytes were isolated by centrifugation followed by red blood cell depletion by lysis, and total RNA was extracted and stored at −80°C prior to mRNA analysis. Serum and urine samples were stored at −80°C prior to protein quantification by ELISA.

### Real-Time Quantitative PCR

Total RNAs from blood leukocytes and human umbilical vein endothelial cells (HUVEC) were extracted with TRIzol (Invitrogen, Saint-Aubin, France) and a commercial kit (Macherey Nagel, Hoerdt, France), respectively. Genomic DNA was removed using DNA-free kit (Applied Biosystems, Life Technologies, Saint Aubin, France) and first-strand reverse transcription (Applied Biosystems) was performed. Real-Time PCR assays were performed on a RotorGene Q (Qiagen, Courtaboeuf, France) following the manufacturer’s recommendations. Human DNA primers were designed using OligoPerfect™ (Invitrogen), QuantPrim (Universität Potsdam, Max-Planck-Gesellschaft) and OligoAnalyser (Integrated DNA Technologies, Inc) with the sequences detailed in Table S1. mRNA expression levels in the samples relative to expression in day 0 were determined with the Pfaffl method (expressed as Relative Fold Change), using ribosomal L19, S9 and RPLPO genes as internal controls. mRNA sST2 levels were assessed using sST2/ST2L ratio (sST2 primer detected a common region of soluble and membrane ST2 while ST2L primer detected only the membrane region).

### Soluble Protein Quantification

HMGB1 (Uscn, Life Science Inc, Euromedex, Strasbourg, France), IL-33 (R&D Systems, Lille, France) and soluble ST2 (sST2) (R&D Systems, France) were assessed in serum, urine and culture cell supernatants using ELISA kits according to the manufacturer’s instructions. The optical density was determined using a microplate reader set to 450 nm (Victor3, Perkin-Elmer, Courtaboeuf, France). HMGB1, IL-33 and ST2s levels in urine samples were also expressed as HMGB1/creatinine, IL-33/creatinine and sST2/creatinine ratios in order to correct for differences in dilution.

### In Vitro Cellular “Ischemia-Reperfusion” Model

Normal primary HUVEC up until passage 4 were cultured in Medium 200+ Low Serum Growth Supplement containing 2% fetal bovine serum (FBS) (Invitrogen, Saint Aubin, France) in a humidified atmosphere at 5% CO_2_ and 37°C. Cellular “ischemia” was achieved by incubating cells in a hermetic chamber containing an hypothermic (4°C) and hypoxic atmosphere: 0% O_2_, 5% CO_2_, and 95% N_2_ (Bactal 2 gaz, Air Liquide, Puteaux, France) during 16 h, in University of Wisconsin solution (UW, Bristol Meyers Squibb, Rueil-Malmaison, France). At the end of the conservation period (H16h), “normothermic reoxygenation” was performed by replacing UW conservation solution by Medium 200+2% FBS for 1 h or 3 h incubation at 37°C in a humidified atmosphere 20% O_2_, 5% CO_2_, and 75% N_2_. Culture supernatants were collected, centrifuged to removed cells, and stored at −80°C prior to soluble protein quantification. In the same times, monolayer cells were collected for RNA analysis.

### PBMC Culture

PBMCs from healthy individuals were plated at 2×10^6^ cells/ml in complete RPMI 1640 medium containing 10% FBS for 3 h, 6 h or 24 h at 37°C in humidified 5% CO_2_ in medium alone or in presence of rhHMGB1 (350 ng/ml, R&D Systems) or rhIL-33 (10 ng/ml, R&D Systems). Then, cells were harvested and stained for flow cytometry analysis, as described below.

### Flow Cytometry

Cells were stained with membrane antibodies diluted in PBS containing 2% FBS and fixed. For *in vitro* experiments, cell viability was evaluated by the LIVE/DEAD® Fixable Near-IR Dead Stain Kit (Invitrogen). Membrane antibodies FITC mouse anti-human CD3, PE mouse anti-human iNKT cell (clone 6B11) and PerCP/Cy5.5 mouse anti-human CD69 were provided by BD Biosciences (San Jose, CA). Appropriate isotype control was used for each marker. Cells were analysed by FACS Canto II™ and FacsDiva™ software (BD, Franklin Lakes, NJ) and data were analysed using FlowJo™ software (version 7.5.5 Treestar, Ashland, OR). CD69 expression was analysed gating on CD3(+)6B11(+) cells, defined as iNKT cells.

### Statistical Analysis

Data are expressed as means ± SEM. HMGB1, IL-33 and sST2 levels in serum and urine samples, and mRNA expression levels in peripheral leucocytes were compared using the Wilcoxon’s or Mann-Whitney test, as appropriate. For the correlation analysis between serum IL-33 levels and duration of cold ischemia, renal function and acute rejection, the Spearman’s test was used. For experiments with HUVEC, statistical data were generated using the one-tailed Mann-Whitney test. Comparisons between the mean fluorescence intensity (MFI) values of CD69 expression levels were performed using the two-tailed Wilcoxon’s test. Differences were considered statistically significant when p<0.05. All statistical analyses were performed using the GraphPadPRISM 5.0 software (GraphPad, La Jolla, CA).

## Results

### Early Release of Alarmins After Renal Transplantation

**Figure 1:**
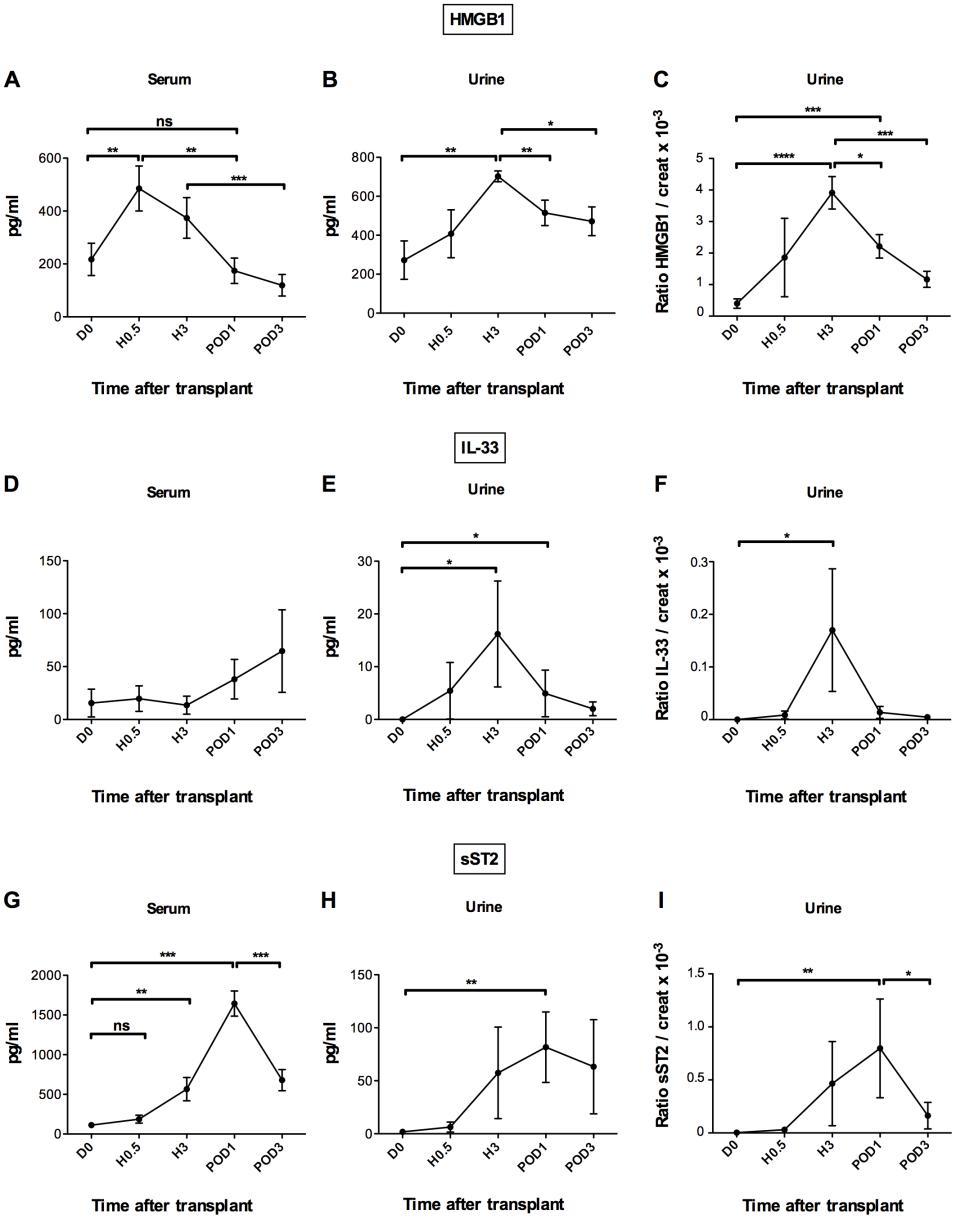
Increased levels of HMGB1, IL-33 and sST2 in serum and urine shortly after renal IRI. HMGB1, IL-33 and sST2 levels were quantified by ELISA in serum and urine of kidney graft recipients (n = 26) before transplantation (D0) as control time, and 30 minutes (H0.5), 3 hours (H3), day 1 (POD1) and day 3 (POD3) after transplantation. Serum, urine and urinary molecule/creatinine ratio levels for HMGB1 (A–C), IL-33 (D–F) and sST2 (G–I). Note that serum samples from only 6 out of 26 transplanted patients contained measurable amounts of IL-33. Data are expressed as means ± SEM. *p<0.05, **p<0.01, ***p<0.001 by Wilcoxon or Mann-Whitney test, as appropriate. ns, no significant.

To investigate the effect of renal IRI on HMGB1 and IL-33 release, we first determined their serum levels. To this end, peripheral blood was taken from patients before and after kidney transplantation, at H0.5, H3 and POD1 and POD3 of reperfusion and assessed by ELISA. Serum HMGB1 was detected before treatment and increased significantly as soon as 30 min after reperfusion (485.3±85.2 pg/ml at H0.5 *vs* 217.1±61.0 pg/ml at D0, p<0.05), returning to baseline levels within 24 hours (Figure 1A). By contrast, IL-33 was barely detectable in the serum of both healthy donors (unpublished data) and patients before transplantation (15.6±13.1 pg/ml). Even though the variations after transplantation did not reach statistical significance (Figure 1D), presumably because IL-33 could only be detected in 6 out of 26 patients (23.0%), we found a significant correlation between these serum levels and cold ischemia time from H0.5 to POD3, which was not the case for HMGB1 (Table 2). Inasmuch as severity of tissue injury can be considered a reliable indicator of cold ischemia, these data support a close relationship between IL-33 release and kidney IRI. Postulating that renal inflammatory injury might be concomitant with local release of alarmins, we further assessed urinary excretion of HMGB1 and IL-33, expressed as alarmin/creatinine ratios to correct for dilution. As shown in Figure 1B and E, urinary levels of both HMGB1 and IL-33 were significantly higher at H3 than before transplantation (702.4±27,6 *vs* 272.2±98,7 pg/ml, p<0.001 and 16.2±10.0 *vs* 0 pg/ml, p<0.05, respectively). The similar expression profiles, whether alarmin levels were normalized against urinary creatinine or not (Figure 1C, F), point to renal endothelial and/or tubular epithelial cells as main cellular origin of HMGB1 and IL-33, as the increase cannot be explained simply by changes in glomerular filtration rate. Lastly, correlations between urinary levels of the two alarmins and cold ischemia time were positive early after reperfusion, although statistical significance was reached only for HMGB1 on POD1 (Table 2). Here again, as for serum levels, a significant positive correlation between urinary IL-33 levels and cold ischemia duration appeared later, on POD3. However, no correlation was found between g001serum or urinary alarmin levels and eGFR or incidence of acute rejection.

**Table 2:** Correlation of serum and urinary alarmin levels with cold ischemia time.

	*Serum*				*Urine*			
	*HMGB1*		*IL-33*		*HMGB1*		*IL-33*	
*Time*	*r*	*p-value*	*r*	*p-value*	*r*	*p-value*	*r*	*p-value*
D0	0.12	0.56	0.4	0.57	0.11	0.73	ND	ND
H0.5	−0.10	0.63	**0.51**	**0.02**	0.39	0.39	0.03	0.93
H3	−0.04	0.85	**0.53**	**0.01**	0.29	0.28	**0.60**	**<0.01**
POD1	0.29	0.14	**0.56**	**<0.01**	**0.50**	**0.05**	0.41	0.06
POD3	0.14	0.49	**0.58**	**<0.01**	0.08	0.74	**0.57**	**<0.01**

POD: Post Operative Day. The correlation coefficient (*r*) is calculated by the non-parametric Spearman’s rank correlation test. A p-value<0.05 was considered significant.

We next analyzed sST2 levels, which are commonly considered a functional signature of IL-33 *in vivo* [24], [25]. A transient increase occurred within 3 hours (556.3±146.4 pg/ml) after reperfusion, reaching a peak at POD1 (1644.0±158.0 pg/ml) compared to D0 (111.7±25.6 pg/ml) (*p*<0.001) and declining thereafter between POD1 and POD3 (679.9±133.3 pg/ml) (Figure 1G). Note that in urine samples kinetic profiles were similar, whether they were expressed as sST2/Cr ratios or as such, without correction for dilution (Figure 1H–I). These overlapping patterns along with the fact that sST2 levels are more than 10-fold higher in serum than in urine are consistent with a major systemic production of this potential decoy receptor. This hypothesis is supported by the early increase of sST2 transcripts in PBMCs, at H0.5 (Figure S1).

Altogether, these data provide additional support to the proposal that IL-33 and HMGB1, which are released early after kidney transplantation, are potential players in IRI.

### Hypoxia/Re-oxygenation-induced Release in vitro of HMGB1 and IL-33

**Figure 2:**
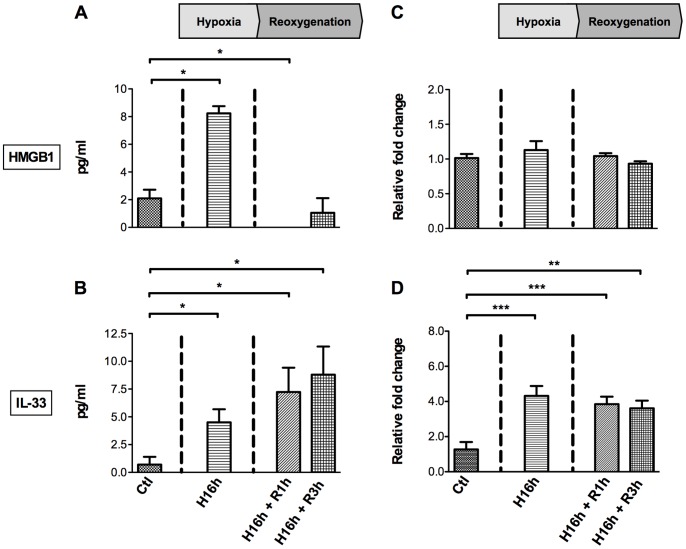
Hypoxia/re-oxygenation-induced release *in vitro* of HMGB1 and IL-33. Confluent (≈95%) monolayer HUVEC were exposed to sixteen hours hypothermia/hypoxia in UW solution (H16h) followed by 1 hour (R1h), or 3 hours (R3h) of re-oxygenation in a new culture medium (Medium 200) at 37°C in 20% O_2_. Confluent (≈95%) monolayer HUVEC were used as controls (Ctl). Early release of HMGB1 and IL-33 by HUVEC in response to in vitro hypoxia/re-oxygenation (A–B). HMGB1 (A) and IL-33 (B) in cell culture supernatants were quantified by ELISA. Increase of IL-33 but not HMGB1 mRNAs in HUVEC in response to *in vitro* hypoxia/re-oxygenation (C–D). Total RNA was extracted from monolayer HUVEC at the indicated time points and expression of HMGB1 (C) and IL-33 (D) mRNAs was quantified by RT-qPCR. Data are expressed as means ± SEM or of fold change relative to D0 and are representative of three separate experiments. *p<0.05, **p<0.01, ***p<0.001 *vs* Ctl by Mann-Whitney test.

To find out whether the early increase in serum and urinary alarmin levels after kidney transplantation originated directly from cell stress during IR, we took advantage of the HUVEC *in vitro* model of hypoxia/re-oxygenation that is widely recognized for mimicking *in vivo* conditions after IR. Moreover HUVEC are well known to express both HMGB1 and IL-33 [26] [27]. To this end, HUVEC were exposed for 16 hours to hypothermic hypoxia. As shown in Figure 2A–B, this treatment led to a marked increase of both HMGB1 (8.2±0.5 *vs* control conditions: 2.1±0.6 pg/ml; *p*<0.05) and IL-33 (4.5±1.2 *vs* control conditions: 0.7±0.7 pg/ml, p<0.05) in supernatants from oxygen-deprived cells, supporting the assumption that cold ischemia is sufficient to promote HMGB1 and IL-33 release. We assessed further whether reperfusion contributed likewise to alarmin increase by subjecting HUVEC for 16 hours to hypothermic hypoxia followed by washing at 1 and 3 hours of normothermic re-oxygenation. This procedure resulted in additional release of IL-33, in clear contrast with HMGB1, which remained close to control values (Figure 2A–B).

As both alarmin and cytokine functions have been attributed to IL-33, we investigated whether the selective and sustained release of IL-33 during the re-oxygenation sequence *in vitro* was in part due to neosynthesis. We found that *IL-33* mRNA levels were significantly increased after the hypoxia sequence and maintained after reperfusion (Figure 2D). By contrast, neither hypoxia nor re-oxygenation affected *HMGB*1 transcription (Figure 2C).

### Upregulation of HMGB1 and IL-33 Receptor Expression in Peripheral Blood Leukocytes from Kidney Graft Recipients

**Figure 3:**
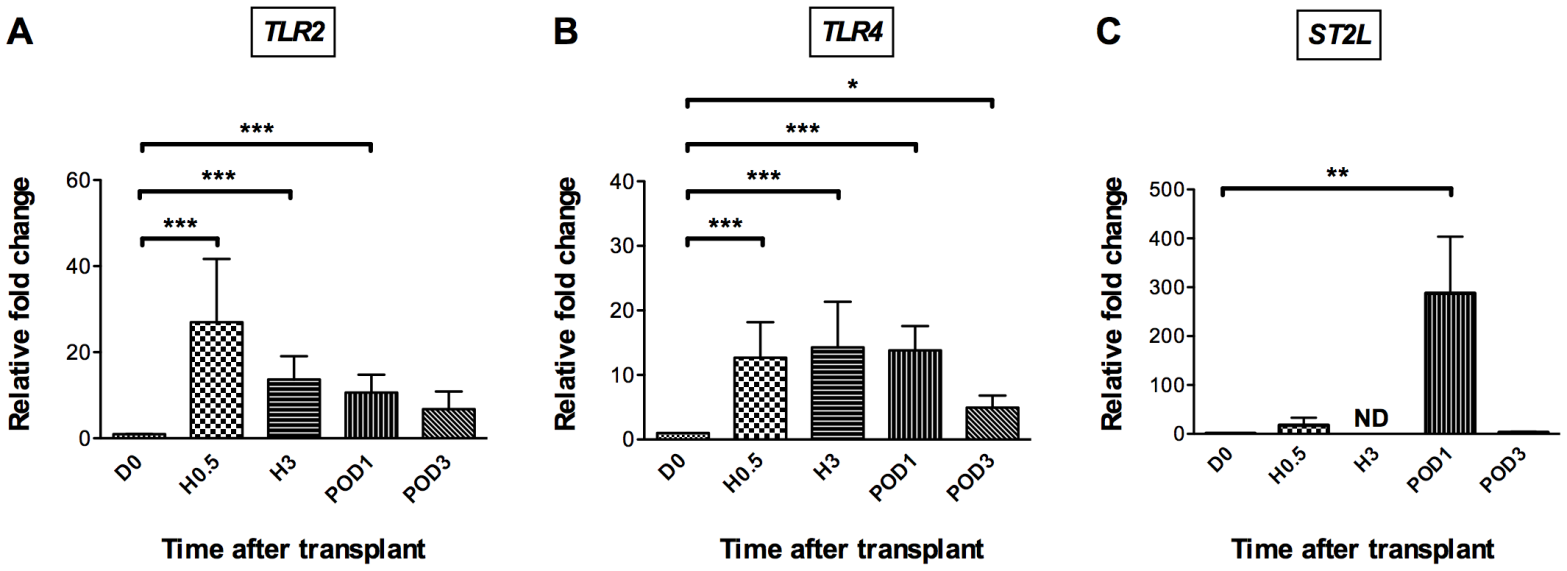
Up-regulation of alarmin-receptor mRNAs in leucocytes after renal IRI. Patient peripheral blood was recovered before transplantation (D0) as control time, and 30 minutes (H0.5), 3 hours (H3), day 1 (POD1) and day 3 (POD3) after transplantation. Total RNA was extracted from leucocytes at the indicated time points and expression of TLR2 (n = 24) (A), TLR4 (n = 25) (B), and ST2L (n = 14) (C) mRNAs was quantified by RT-qPCR. Results are expressed as means ± SEM of fold change relative to D0. *p<0.05, **p<0.01, ***p<0.001 *vs* D0 by Wilcoxon test. ND: not done.

The early release of HMGB1 and IL-33 *in vivo* after kidney reperfusion along with enhanced secretion by HUVEC in response to hypoxia/re-oxygenation conditions *in vitro* led us to address the possible role of these two alarmins as innate-immune mediators during IRI. To this purpose, we determined whether in our kidney graft recipient cohort HMGB1 and IL-33 release was accompanied by increased transcription of their specific receptors, namely TLR2, TLR4, and ST2L in blood leukocytes, using real-time RT-qPCR (Figure 3A–C). While none of these transcripts could be reliably detected before transplantation, a positive signal for *TLR2, TLR4* and *ST2L* mRNA appeared as soon as 30 min after reperfusion, although statistical significance was reached only for *TLR2* and *TLR4*. At POD1, all three receptor transcripts increased significantly over pre-transplantation levels, reaching a peak for *TLR4* and *ST2L* and dropping to near baseline levels at POD3 for all three. These results confirm previous evidence for increased HMGB1 receptor transcription in peripheral blood leukocytes during IR [16] and extend this observation to the specific IL-33 receptor ST2L.

### Early Activation of iNKT Cells after Renal Transplantation: A Potential Role for the Alarmin IL-33

**Figure 4:**
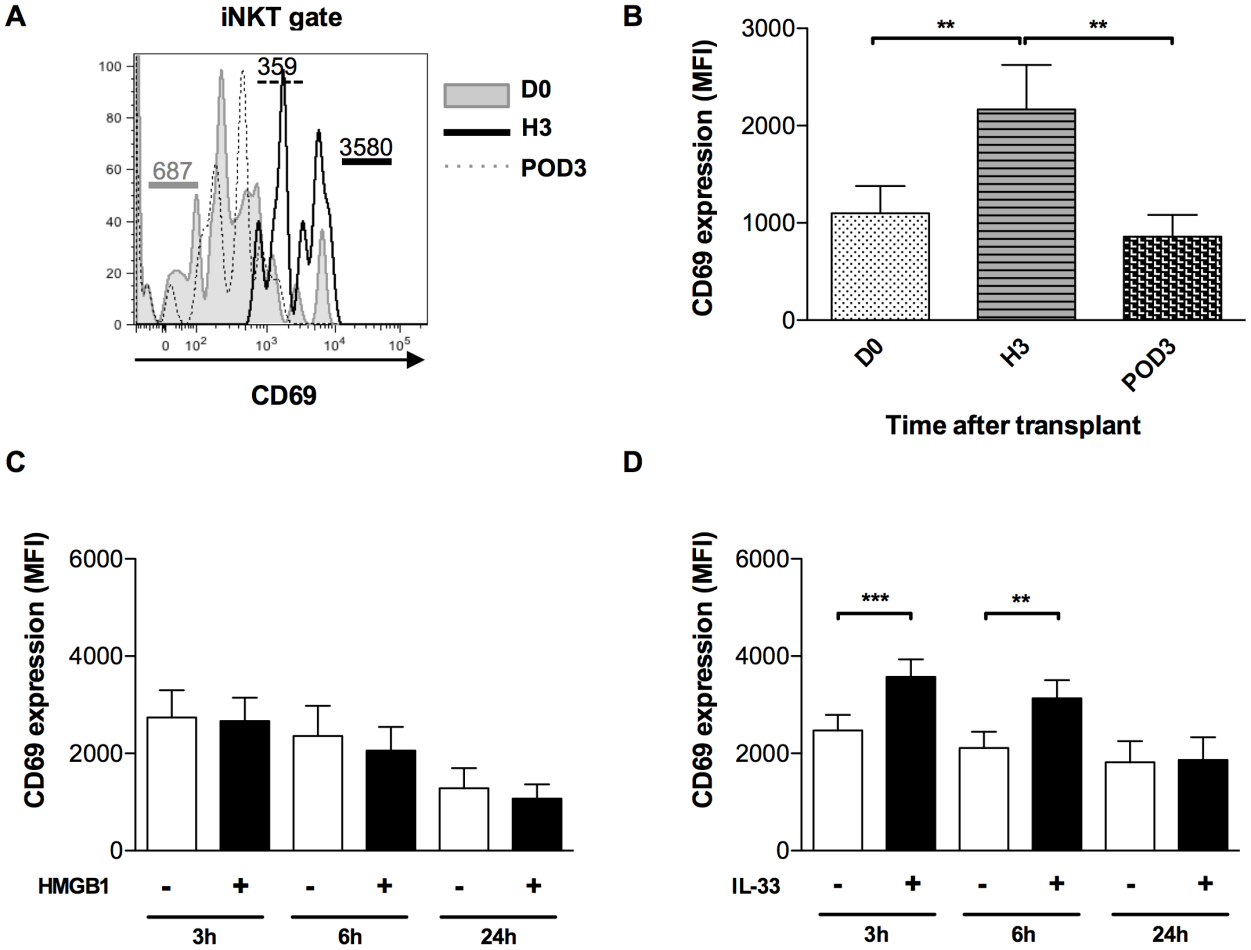
Early activation of iNKT cells after renal IRI: a potential role for IL-33. (A, B) PBMCs from kidney graft recipients were recovered before transplantation (D0), and 3 hours (H3) and day 3 (POD3) after transplantation. They were membrane-labelled with anti-CD3-FITC, anti-iNKT-PE 6B11 clonotype, and anti-CD69-PerCP/Cy5.5. CD69 analysis was performed by flow cytometry gating on CD3(+)6B11(+) cells, defined as iNKT cells: (A) Flow cytometry plot showing expression profiles of surface marker CD69 on iNKT cells *ex vivo* from one representative patient at D0 (filled histogram), H3 (bold line) and POD3 (dotted line). Numbers indicate MFI of CD69 expression on iNKT lymphocytes. (B) Mean Fluorescence Intensity (MFI) of CD69 expression on iNKT lymphocytes from the patient cohort (n = 16 at D0, H3, and POD3). (C, D) PBMCs from healthy adult donors (n = 6) were cultured with (black columns) or without (white columns) HMGB1(C) or IL-33 (D) for 3, 6 or 24 hours of culture. MFI of CD69 expression on iNKT lymphocytes was analysed by Flow cytometry as described in (A, B). Data are expressed as means ± SEM. **p<0.01, ***p<0.001 by Wilcoxon test.

Previous reports have shown that iNKT cells are activated rapidly during experimental renal IRI. Indeed, these innate-like cells are among the first to infiltrate the kidney after IR onset in mice [28]. Moreover, mouse and human iNKT cells express ST2L and can be activated *in vitro* and *in vivo* by IL-33 [29] [30] [31]. To assess whether in our clinical setting IL-33 released during IRI could actually target iNKT cells, we analyzed the early activation marker CD69. As shown in Figure 4A–B, peripheral blood iNKT cells were promptly and transiently activated after reperfusion, in terms of CD69 surface expression which was upregulated 3 hours after transplantation and declined thereafter. In the same line of evidence, we addressed the question whether HMGB1 and/or IL-33 contributed directly to iNKT-cell activation during IR, by exposing PBMCs from healthy donors *in vitro* to each of the two alarmins. Exogenous IL-33 increased CD69 expression within only 3 hours, similarly to the kinetics obtained during IR, whereas HMGB1 had no such effect (Figure 4C–D). It can therefore be concluded that IL-33 is a potential innate-immune mediator during kidney IRI in humans.

## Discussion

A number of animal studies [12] [31] [32] designate HMGB1 and IL-33 as putative endogenous molecules triggering sterile inflammatory signals associated with IRI in humans. However, to our knowledge, IL-33 has not yet been investigated during renal transplantation in humans [16].

Here we show that both HMGB1 and IL-33 are significantly increased in the urine of kidney graft recipients within only 3 hours post-transplantation, which suggests that they are generated by events initiated by IR. Furthermore, we provide evidence that this early release of alarmins causes prompt activation of leukocyte populations belonging to the first line of defense of the immune system. Indeed, we found that *TLR2-4* and *ST2L* mRNA levels were rapidly increased in leukocytes after reperfusion, consistent with a DAMP-mediated danger signal acting at the systemic level. This increase occurred concomitantly with IL-33 release and activation of iNKT cells in terms of CD69 upregulation, suggesting that this leukocyte population is targeted by IL-33 in patients having received a kidney graft. This conclusion is in agreement with our *in vitro* data, which establish that exogenous IL-33 can activate iNKT cells from healthy individuals. It concurs likewise with our previous evidence in mouse models showing that IL-33 can target iNKT cells *in vivo* [33].

These results raise the question of the cellular origin of HMGB1 and IL-33. The overlapping profiles of HMGB1 production in serum and urine are consistent with a passive release from necrotic tubular and epithelial cells, as described in mouse models [12]. Moreover, HMGB1 expression has been reported in tubular cells from implantation biopsies of deceased donors only, suggesting a relationship with hypoxic cell damage [16]. Similarly to HMGB1, urinary excretion of IL-33 occurred rapidly upon transplantation, arguing in favor of a release by ischemic tubular cells, although a possible contribution of vascular endothelial cells cannot be excluded. Indeed, in a mouse model of acute kidney injury (AKI), sharing the occurrence of cell damage with kidney IRI, immunofluorescence staining revealed IL-33 expression on the endothelial surface of blood vessels, as well as in glomeruli and in peritubular capillaries [21].

The involvement of HMGB1 in renal IRI is supported by studies in both animal models and clinical situations. TLR2 and TLR4 have been reported for being upregulated by ischemic AKI in renal tubular as well as endothelial cells of mice [34] [35] and for mediating kidney IRI in humans [12] [15]. Moreover, the capacity of HMGB1 to activate the innate immune system early after transplantation depends on these two TLRs [9] [12] [36]. Following human kidney transplantation, tubular expression of TLR4 was higher in kidneys from deceased than from living donors, suggesting a control by ischemic stress intensity [16]. Conversely to HMGB1, the consequences of IL-33 release during kidney injury have not been investigated so far, excepting one study in which administration of sST2 was shown to protect from cisplatin-induced AKI, indicating that in this model it behaved like a decoy receptor to neutralize endogenous IL-33 [21]. In the same line of evidence, acquired even before IL-33 was identified as ST2 ligand, administration of sST2-Fc fusion protein in mice was shown to attenuate inflammation in intestinal ischemia reperfusion [37], in support of the alarmin function of IL-33 during IRI. Our evidence for high circulating levels of soluble ST2 during renal transplantation suggests that the IL-33/ST2 axis can also mediate the systemic inflammatory reaction after renal IR, as reported in a variety of diseases [38]. Lastly, in accordance with the alarmin concept, one may speculate that IL-33 release could promote the recruitment of neutrophils and/or CD4(+) T cells within the graft [21]. This hypothesis deserves particular attention, knowing that IL-33 promotes rapid activation of iNKT cells (Figure 4D) [29] [30], which are widely acknowledged for their capacity to recruit neutrophils during experimental kidney IRI [28].

It remains to be established whether alarmins could become instrumental as predictive biomarkers of IRI. Although HMGB1 release reportedly reflected the extent of ischemic injury in a rat model of liver transplantation [39], its prognostic value for the outcome of human solid organ grafts is still uncertain. The correlation between cold ischemia duration and blood IL-33 levels, 30 min after reperfusion, suggests a close link between alarmin release and intensity of renal cell damage. The failure to detect IL-33 in more than 50% of the sera analyzed could be a consequence of the autocrine/paracrine functions of this alarmin, which is locally produced and more frequently detected in peripheral fluids [40] [41]. In accordance with this view, urinary IL-33 was detected in 90% of graft recipients, by contrast with its virtual absence in healthy donors (unpublished data). This observation suggests that the production of IL-33 could be restricted to kidney transplantation, which would be an asset as a biomarker for renal IRI.

Since prolonged cold ischemia is associated with delayed graft function and graft loss [42], the development of new prognostic indicators for the follow up of ischemia, such as IL-33 and/or sST2 is worthwhile and should be evaluated in an extensive study based on our preliminary results.

Although the respective impact of IL-33 and HMGB1 on renal IR remains to be delineated *in vivo*, our *in vitro* model of hypothermic/hypoxic cellular stress, reveals that the kinetics and mode of release are not quite the same. Thus, IL-33 is increased during both hypoxia and reperfusion sequences, while HMGB1 is only induced during hypoxia, a difference that could result from selective induction of IL-33 transcription. As a corollary *in vivo*, IL-33 release was maintained for 1–3 days after transplantation, presumably due to further synthesis during the reperfusion sequence. Hence, we postulate that IL-33 may act both as an alarmin and a conventional multi-function cytokine during human kidney transplantation [19]. For this reason, it is actually difficult to predict whether it exerts a deleterious or a beneficial effect on renal transplantation. Recently, Sakai *et al*. [23] reported that the early release of IL-33 during liver IR in mice was protective, as it reduced hepatocyte cell death by promoting the expression of anti-apoptotic genes. Moreover, in a mouse model of cardiac transplantation, IL-33 was found to prolong allograft survival, potentially via a Th2 immune deviation [43] [44]. Lastly, Turnquist *et al*. reported the capacity of IL-33 to expand functional Foxp3+ Tregs in murine cardiac allograft [45]. Altogether, these results are consistent with dual functions of IL-33 in solid organ transplantation.

In summary, this study reveals an impact of IRI on HMGB1 and IL-33 alarmin release in human renal transplantation and provides evidence in favor of the concept that kidney IR can be assimilated to an alarmin-mediated inflammatory response. Whether alarmin signaling pathways are a potential therapeutic target to neutralize the innate inflammatory cascade in renal IRI remains to be investigated.

## Supporting Information

Figure S1 **Up-regulation of sST2 mRNAs in PBMCs after renal IRI.** Human PBMCs were recovered before transplantation (D0) as control time, and 30 minutes (H0.5), 3 hours (H3), day 1 (POD1), and day 3 (POD3) after transplantation. Total RNA was extracted from PBMCs at the indicated time points and expression of sST2 mRNAs was quantified by RT-qPCR. Results are expressed as means ± SEM (n = 22) of fold change relative to D0. *p<0.05, **p<0.01, ***p<0.001 *vs* D0 by Wilcoxon test.
(TIF)

Table S1 **Primers used for real-time RT-PCR in human blood leucocytes.**
(DOCX)
