# Supplementary figures and images for: The Alarmin Concept Applied to Human Renal Transplantation: Evidence for a Differential Implication of HMGB1 and IL-33
Source: PLoS One. 2014 Feb 20;9(2):e88742. doi: 10.1371/journal.pone.0088742 (PMC3930579; doi:10.1371/journal.pone.0088742)

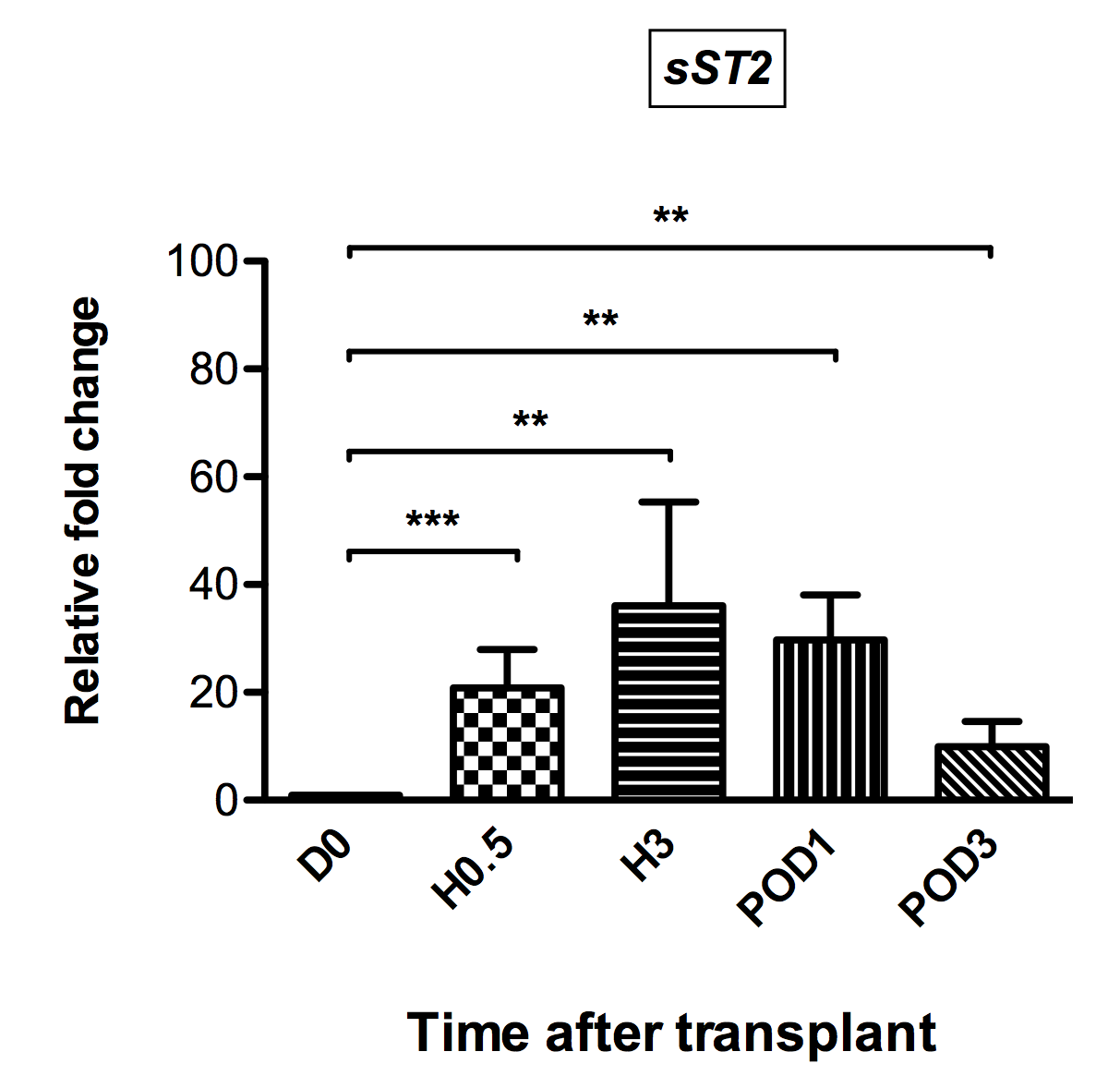

Supplement: Figure S1 — Up-regulation of sST2 mRNAs in PBMCs after renal IRI. Human PBMCs were recovered before transplantation (D0) as control time, and 30 minutes (H0.5), 3 hours (H3), day 1 (POD1), and day 3 (POD3) after transplantation. Total RNA was extracted from PBMCs at the indicated time points and expression of sST2 mRNAs was quantified by RT-qPCR. Results are expressed as means ± SEM (n = 22) of fold change relative to D0. *p<0.05, **p<0.01, ***p<0.001 vs D0 by Wilcoxon test. (TIF) [file pone.0088742.s001.tif]
