# Supplementary material for: The Alarmin Concept Applied to Human Renal Transplantation: Evidence for a Differential Implication of HMGB1 and IL-33
Source: PLoS One. 2014 Feb 20;9(2):e88742. doi: 10.1371/journal.pone.0088742 (PMC3930579; doi:10.1371/journal.pone.0088742)
Supplement: Table S1 — Primers used for real-time RT-PCR in human blood leucocytes. (DOCX) [file pone.0088742.s002.docx]

**Table S1**: Primers used for real-time RT-PCR in human blood leucocytes

| **Target** |  | **Primer Sequence** |
| --- | --- | --- |
| RPL19 | Forward primer | 5’- TGGCAAGAAGAAGGTCTGG -3’ |
|  | Reverse primer | 5’- TCGAGCCCGGGAATG -3’ |
| RPLPO | Forward primer | 5’- CAGGGCGACCTGGAAGT -3’ |
|  | Reverse primer | 5’- TCTGCTTGGAGCCCACA -3’ |
| RPS9 | Forward primer | 5’- TGCTGACGCTTGATGAGAAG -3’ |
|  | Reverse primer | 5’- CGCAGAGAGAAGTCGATGTG -3’ |
| sST2 | Forward primer | 5’-Aaatttattgtcctaccattgacctc -3’ |
|  | Reverse primer | 5’-tgacttgtgcgccctgt -3’ |
| ST2L | Forward primer | 5’- TGCCTGCTGTGATGTGCAAAGG -3’ |
|  | Reverse primer | 5’- CCAGCTAGGAGAAGTCAGGAAAGC -3’ |
| TLR2 | Forward primer | 5’- GCCTCTCCAAGGAAGAATCC -3’ |
|  | Reverse primer | 5’- TCCTGTTGTTGGACAGGTCA -3’ |
| TLR4 | Forward primer | 5’- AAGCCGAAAGGTGATTGTTG -3’ |
|  | Reverse primer | 5’- CTGAGCAGGGTCTTCTCCAC -3’ |
| IL-33 | Forward primer | 5’- GAAAATCCCAACAGAAGGCCA -3’ |
|  | Reverse primer | 5’- GCCTTTTGGTGGTTTCTCTCCT -3’ |
| HMGB1 | Forward primer | 5’- AAAGCGGACAAGGCCCGTTAT -3’ |
|  | Reverse primer | 5’- AAGAGGAAGAAGGCCGAAGGAG -3’ |
